# Novel Indirect Calorimetry Technology to Analyze Metabolism in Individual Neonatal Rodent Pups

**DOI:** 10.1371/journal.pone.0006790

**Published:** 2009-08-27

**Authors:** Jesus F. Dominguez, Lixin Guo, Marco A. Carrasco Molnar, Antonio Ballester Escobedo, Taylor Dunphy, Trent D. Lund, Jack E. Turman

**Affiliations:** 1 Center for Premature Infant Health and Development, Keck School of Medicine, University of Southern California, Los Angeles, California, United States of America; 2 Division of Biokinesiology and Physical Therapy, School of Dentistry, University of Southern California, Los Angeles, California, United States of America; 3 Department of Endocrinology, Beijing Hospital, Beijing, China; 4 Panlab, S.L./Harvard Apparatus, Barcelona, Spain; 5 Stoelting, Inc., Chicago, Illinois, United States of America; 6 Departments of Cell and Neurobiology, and Pediatrics, Keck School of Medicine, University of Southern California, Los Angeles, California, United States of America; Universidad Europea de Madrid, Spain

## Abstract

**Background:**

The ability to characterize the development of metabolic function in neonatal rodents has been limited due to technological constraints. Low respiratory volumes and flows at rest pose unique problems, making it difficult to reliably measure *O_2_* consumption, *CO_2_* production, respiratory quotient (*RQ*), and energy expenditure (*EE*). Our aim was to develop and validate a commercial-grade indirect calorimetry system capable of characterizing the metabolic phenotype of individual neonatal rodents.

**Methodology/Principal Findings:**

To address this research need, we developed a novel, highly sensitive open-circuit indirect calorimetry system capable of analyzing respiratory gas exchange in a single neonatal rodent pup. Additionally, we derived an equation from known metabolic relationships to estimate inlet flow rates, improving the efficiency of data collection. To validate the neonatal rodent indirect calorimetry system and evaluate the applicability of the derived equation for predicting appropriate flow rates, we conducted a series of experiments evaluating the impact of sex, litter size, time of day (during the light phase), and ambient temperature on neonatal rat metabolic parameters. Data revealed that the only metabolic parameter influenced by litter size is a neonatal rat's *RQ*, with rat pups reared in a small litter (5 pups) having lower *RQ's* than rat pups reared in either medium (8 pups) or large (11 pups) litters. Furthermore, data showed that ambient temperature affected all metabolic parameters measured, with colder temperatures being associated with higher *CO_2_* production, higher *O_2_* consumption, and higher energy expenditure.

**Conclusion/Significance:**

The results of this study demonstrate that the modified Panlab Oxylet system reliably assesses early postnatal metabolism in individual neonatal rodents. This system will be of paramount importance to further our understanding of processes associated with the developmental origins of adult metabolic disease.

## Introduction

The developmental origins of adult disease hypothesis (often referred to as the Barker hypothesis), asserts that perturbations occurring during the perinatal period can have profound effects on the development of metabolism and other physiologic processes in adulthood [1], [2]. Understanding the development of metabolic function in individual neonatal rodent pups becomes of paramount importance as these are often the models of choice when studying the etiology of, and interventions for, fetal programmed human diseases that include obesity, diabetes, hypertension, and the metabolic syndrome. Being able to characterize the metabolic phenotype of transgenic and knockout models of disease would greatly contribute to our understanding of many pathophysiologic processes and provide a foundation for developing effective treatment strategies.

Until recently, technological constraints have made it difficult to reliably collect measurements of oxygen consumption (*VO_2_*, in ml/min/kg), carbon dioxide production (*VCO_2_*, in ml/min/kg), respiratory quotient (*VCO_2_/VO_2_*, or *RQ*) and energy expenditure (*EE*, in kcal/day/kg) via indirect calorimetry in individual neonatal rat pups, thus limiting our understanding of neonatal metabolic development. In large part, this is due to the low air flows that are required to accurately sample the small volumes of respiratory gases exchanged by these animals during the resting state [3], [4]. The analysis is further complicated by the need to allow for an adequate steady-state equilibration time followed by an appropriate sampling period while minimizing the stress incurred by the pup during periods of prolonged maternal deprivation [5]. Finally, developing a means of predicting the appropriate inlet air flow rates is essential to overcome the limitations mentioned above and minimize the need for prolonged trial and error methodology. Analysis of neonatal rodent metabolism necessitates that indirect calorimetry systems have components sensitive enough to ensure that 1) low flows are delivered accurately to allow sufficient time for chamber gas equilibration and do not wash out the small changes created by the rat pup, 2) room air is prevented from entering the chamber through the pressure equalization port so as not to obscure the true values of the gases contained within, and 3) relatively small changes in the chamber *O_2_* and *CO_2_* concentrations are detected by its sensing components. These considerations require the incorporation of special low-flow regulators and highly sensitive gas analyzers into the metabolic analysis apparatus.

We have partnered with Panlab, S.L.\Harvard Apparatus (Barcelona, Spain) and Stoelting, Inc. (Chicago, IL) in order to construct a novel metabolic analysis system suitable for evaluating metabolism in a single neonatal rodent preparation. The Panlab Oxylet System (Panlab, S.L., Barcelona, Spain) is a commercially available system that uses open-circuit indirect calorimetry to study metabolism in adolescent and adult laboratory rodents (specifications available from the manufacturer). Here we describe a modification of this system and a method for estimating appropriate air flow rates based on animal weight and expected gas concentrations that will yield reliable results in a single neonatal rodent preparation. This new system is the first-commercial grade open-circuit indirect calorimetry system for neonatal rodents, and herein we present a series of studies conducted to validate the system and evaluate its efficacy in detecting differences in metabolic parameters from known or expected sample outcomes.

## Methods

All procedures were approved by the University of Southern California's Institutional Animal Care and Use Committee (IACUC) in accordance with National Institutes of Health (NIH) guidelines. To validate the neonatal rodent indirect calorimetry system and evaluate the applicability of the derived equation for predicting appropriate flow rates, we conducted a series of experiments evaluating the impact of sex, litter size, time of day (during the light phase), and ambient temperature on neonatal rat metabolic parameters. Pregnant rats were delivered to our facility on gestational day 15. Pups were housed in a single cage with their dams. The housing facility was maintained at an ambient temperature of 23°C with a standard 12-hour light/12-hour dark cycle and the dams were allowed ad libitum access to food and water while the pups were allowed to suckle freely.

**Experiment 1.** Fourteen Sprague Dawley rat pups born to three different timed pregnant dams (Charles River Laboratories, Inc., Wilmington, MA) were used to evaluate the ability of the system to detect the influence of sex, litter size, and time of day (during the light phase) on early postnatal metabolic parameters. On postnatal day (P) 1, one litter was culled to 5 pups (termed small [*SML*] litter in the text), one litter was culled to 8 pups (termed medium [*MED*] litter in the text), and one litter was culled to 11 pups (termed large [*LRG*] litter in the text). The sample sizes surveyed with indirect calorimetry for each litter group were; *SML* (n = 5), *MED* (n = 4), and *LRG* (n = 5). Because of the direct relationship between metabolic parameters and growth outcomes, all the pups had body weight, snout-to-rump (*SR*) length, and snout-to-occiput (*SO*) length measures recorded immediately prior to each testing trial in the metabolic chamber. This was done in order to ensure that growth patterns observed in our study did not differ significantly from normal growth patterns observed in early postnatal rats reared in small, medium, and large litters [6].

**Experiment 2.** Sixteen Sprague Dawley rat pups born to two timed pregnant dams (Charles River Laboratories, Inc., Wilmington, MA) were used to evaluate the ability of the system to detect the influence of ambient temperature on early postnatal metabolic parameters. On postnatal day (P) 1, each litter was culled to 8 pups. The pups were then randomly assigned to one of three experimental groups to be surveyed with indirect calorimetry at different chamber ambient temperatures; a 25°C ambient temperature group (*25°C*, n = 5), a 30°C ambient temperature group (*30°C*, n = 5), and a 34°C ambient temperature group (*34°C*, n = 6).

### Respiratory Gas Collection and Analysis

**Figure 1:**
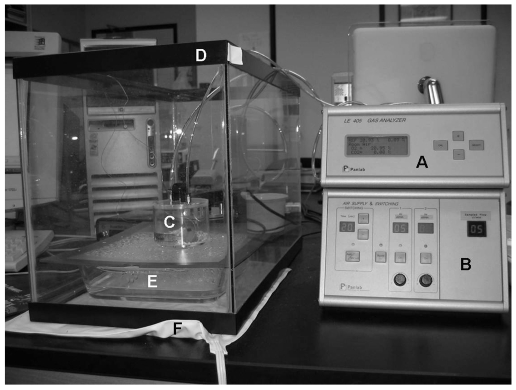
Oxylet Neonatal Indirect Calorimetry System. To collect metabolic data, the Panlab Oxylet neonatal rodent indirect calorimetry system prototype was used in conjunction with associated apparati. The following components are identified: (A) LE 405 gas analyzer, (B) LE 400 Air Supply and Switching unit, (C) 215 ml metabolic chamber with bedding material, (D) 10-gallon aquarium, (E) Plexiglas sheet over water-filled Pyrex pan, and (F) variable-control heating pad.

Prior to data collection on each of the experimental days, the Oxylet indirect calorimetry system was calibrated using certified gas cylinders (Gilmore Liquid Air Co., South El Monte, CA) containing 50% *O_2_*/5% *CO_2_*/45% *N_2_* (high point calibration) and 20% *O_2_*/0% *CO_2_*/80% *N_2_* (low point calibration) per manufacturer's instructions. A variable control heating pad was placed underneath a 10-gallon aquarium containing a Pyrex pan filled with distilled water. A cylindrical, clear, Plexiglas metabolic chamber, containing a small amount of bedding material from the pup's home nest, was placed on a plexiglass sheet over the Pyrex pan (Figure 1). For *Experiment 1*, the ambient chamber temperature was maintained at 28°C, a temperature below thermoneutral but not considered to be stressful to the pups [7]. For *Experiment 2*, the ambient temperature was maintained at one of the experimental conditions (*25°C*, *30°C*, or *34°C*). Because the Oxylet prototype utilized an open-circuit design, the chamber temperature could be constantly monitored by passing a fine-wire microprobe thermocouple (Physitemp BAT-12, Physitemp Instruments Inc., Clifton, NJ) through the chamber lid's pressure equalization port. A pup's weight was entered into the derived inlet flow rate equation (see below) to estimate the appropriate value. Individual pups were placed in the metabolic chamber and allowed to acclimate for 15 minutes prior to data collection. During this period, room air was allowed to flow through the chamber.

After acclimation, respiratory gas analysis was carried out utilizing Metabolism^®^ software (Panlab, S.L., Barcelona, Spain) running on a HP Compaq Pentium 4 computer (Hewlett-Packard Company, Palo Alto, CA). The data collection paradigm consisted of 1) a 15-minute acclimation period during which time room air was flowing through the metabolic chamber, 2) a 1-minute room air reference sampling period, and 3) a 45-minute chamber air sampling period. This paradigm yielded enough time for the small volumes of gases exchanged by the rat pup to reach equilibrium within the chamber, accommodating the inherently slow sampling process associated with low inlet and sampling flow rates. The mean values of *VO_2_*_,_ *VCO_2_*, *RQ*, and daily *EE* over the 45-minute chamber sampling period were then calculated by the Metabolism^®^ software. The total time a pup spent away from its dam was limited to 1 hour. These time parameters were selected to minimize the pup's separation from its dam, as we did not want stress to confound the results [5]. Metabolic data were collected from each pup on alternate days beginning on P2 and ending on either P6 (*Experiment 2*) or P14 (*Experiment 1*). Each pup was tested at the same time of day on each testing day, and cohorts were divided into an AM group (tested between 8:00 A.M. and 12:00 P.M., and a PM group (tested between 1:00 P.M. and 5:00 P.M.) to explore the possible influence of time of day (during the light phase) on metabolic parameters (*Experiment 1*).

Initially, a chamber volume of 215 milliliters (chamber *r* = 3.7 cm and *h* = 5.0 cm) was used for pups between P2-10 to minimize the amount of airspace around the pup and reduce the time required for gas equilibration. A chamber volume of 550 milliliters (chamber *r* = 5.75 cm and *h* = 5.3 cm) was used from P12–14 to accommodate the physical dimensions of the growing pups. Additionally, even at airflow rates of 225–250 ml/min (the upper limit of the prototype pump flow rate), the %*O_2_* and %*CO_2_* values could not be optimized in the smaller chamber. Thus, the larger chamber volume was required to maintain the chamber %*O_2_* and %*CO_2_* values within the prescribed ranges. As the pups grew during the course of the experiment, the calculated inlet flow rate was increased on the LE 400 unit. The sampling flow rate leading to the gas analyzer was always maintained below the inlet flow rate to avoid overrunning the inlet flow rate, as this could introduce room air into the chamber via the pressure equalization port and confound the measured gas values.

By utilizing an open-circuit system that pulled air into the chamber under slightly negative pressure to maintain a constant flow rate, the influence of the pup's physical activity on internal chamber pressure (and thus, on the ability to measure true *VO_2_*) was avoided. Nevertheless, because activity level significantly influences metabolism, an activity scoring scale was adopted to assess the pup's gross activity during metabolic data collection for Experiment 1. This was facilitated by the use of a Plexiglas chamber that permitted direct observation of the pups. Gross activity was operationalized and scored in the following manner: 0 = no movement, 1 = head movement only, 2 = forepaw movement only, 3 = head and forepaw movement only, 4 = hindlimb movement only, 5 = head and hindlimb movement only, and 6 = head, forepaw, and hindlimb movement. The scoring paradigm consisted of assessing activity for 1 minute and recording the modal score. This was repeated at 5-minute intervals and a mean activity score was recorded for each rat during each entire metabolic analysis session.

### Oxylet Neonatal Prototype

To construct the Oxylet neonatal prototype, the sampling pump of the Oxylet LE405 gas analyzer was replaced with a more sensitive and stable model and the flow regulators and flow meters of the Oxylet LE 400 air supply and switching unit were replaced with more sensitive components. The Oxylet system utilizes a laser absorption *O_2_* sensor. A tunable laser source with a narrow emission is tuned to the peak of a spectral line of the molecule under study and the absorption of the light from the source is measured. The absorption will depend only on the width of the laser emission line and height of the molecular spectral line. The absorption is instantaneous once the laser beam hits a molecule. Unlike systems using older technology, the measurement process is rapid and is only dependent on the time it takes the laser to travel through the measurement chamber instead of being influenced by the speed of chemical reactions or the mechanical rearrangement of molecules. This technology affords the system significant robustness and allows for the fine tuning needed to reliably measure the metabolic activity of individual neonatal rats. In combination with a *CO_2_* sensor using infrared spectroscopy technology and special polymers, the influence of air humidity on the measurement of metabolic parameters is minimized. This is of particular importance in the evaluation of respiratory metabolism in animals enclosed within a chamber. These components represented a significant improvement over older technology that included the *CO_2_* and water traps of closed-circuit systems. Furthermore, the real-time measurement of *VO_2_* and *VCO_2_* allowed for the assessment of the *RQ*.

As a necessary feature, a separate flow meter was added to the prototype in order to measure the sampling pump flow rate and insure that it remained safely below the inlet flow rate. This prevented the sampling flow rate from overrunning the inlet flow rate and introducing room air into the chamber via the pressure equalization port and confounding the measured gas values. The incorporation of these modifications allowed for fine adjustment of inlet and sampling flows within a range of approximately 0–250 milliliters/minute (compared with a range of 150–2,000 milliliters/minute in the existing adult system).

### Estimation of Inlet Air Flow Rate

Calculation of appropriate inlet flow rates for each pup began with consideration of the formulas used by the Metabolism^®^ software to calculate *VO_2_* and *VCO_2_*:
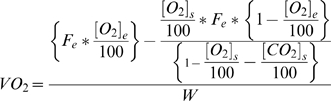
and
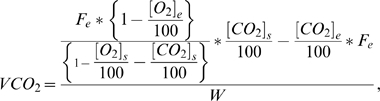
where: 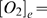
oxygen concentration (room air) flowing into the cage


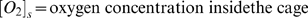



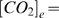
carbon dioxide concentration (room air) flowing into the cage


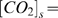
carbon dioxide concentration inside the cage


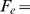
airflow entering the cage


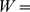
weight of the animal in kg


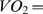
oxygen consumption


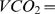
carbon dioxide production.

The *VO_2_* and *VCO_2_* equations were then inverted in order to estimate an inlet flow rate that would yield a specific [*O_2_*]_s_ value for a given *VO_2_*. Isolating [*CO_2_*]*_s_* from the *VCO_2_* equation yields:



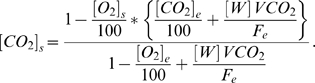



Substituting this value into the *VO_2_* equation gives:
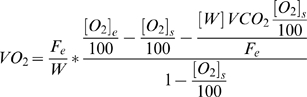



Utilizing the known *RQ* equation
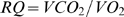
and solving for *F_e_* yields:



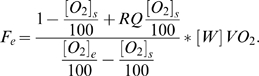



This equation was then used to estimate an appropriate inlet flow rate for each pup. The final estimation of *F_e_* takes into consideration the difference between [*O_2_*]*_e_* and [*O_2_*]*_s_* such that: 1) it is sufficiently different to allow the gas analyzer to detect it and 2) it does not adversely affect the animal's respiration. In general, this difference should be between 0.5–1.0% to yield reliable results [3]. As an initial estimate, the expected *VO_2_*'s for 6 to 7-day-old rat pups tested at different ambient temperatures were obtained from the literature [4], [7]–[8], although there was considerable variation in methodology and reported values. Because the neonatal rat's diet consists of its mother's milk which is high in fat, the resting *RQ* was predicted to be 0.79 [8]–[10]. Finally, the flow rate should be sufficient to maintain the difference between [*CO_2_*]_e_ and [*CO_2_*]_s_ within the range of 0.5–0.9% to allow for detection while maintaining the chamber ambient level below 1.0%, as this is usually considered the upper limit at which rebreathing of *CO_2_* induces hyperventilation [3], [11].

### Data Analysis

All data were analyzed utilizing SPSS v13 software. Outcome measures for this study included body weight, *SR* length, *SO* length, *VO_2_, VCO_2_, RQ, and EE*. Two-way mixed design ANOVAs were conducted using either: sex (male, n = 8, female, n = 6), time of day (AM, n = 7, PM, n = 7), litter size (*SML*, n = 5, *MED*, n = 4, and *LRG*, n = 4), and chamber ambient temperature (*25°C*, n = 5, *30°C*, n = 5, and *34°C*, n = 6) as between subject factors, and testing day (P2–P6 or P2–14, alternate days) as the within subjects factor. Post testing of the between subjects factors was performed using Tukey HSD. Post testing of testing day×litter size interaction was performed using individual one-way ANOVA's with associated Tukey HSD. Only statistically significant differences (p<0.05) or trends (0.05<p<0.09) were reported and discussed. All results were expressed as means±SEM.

## Results

**Figure 2:**
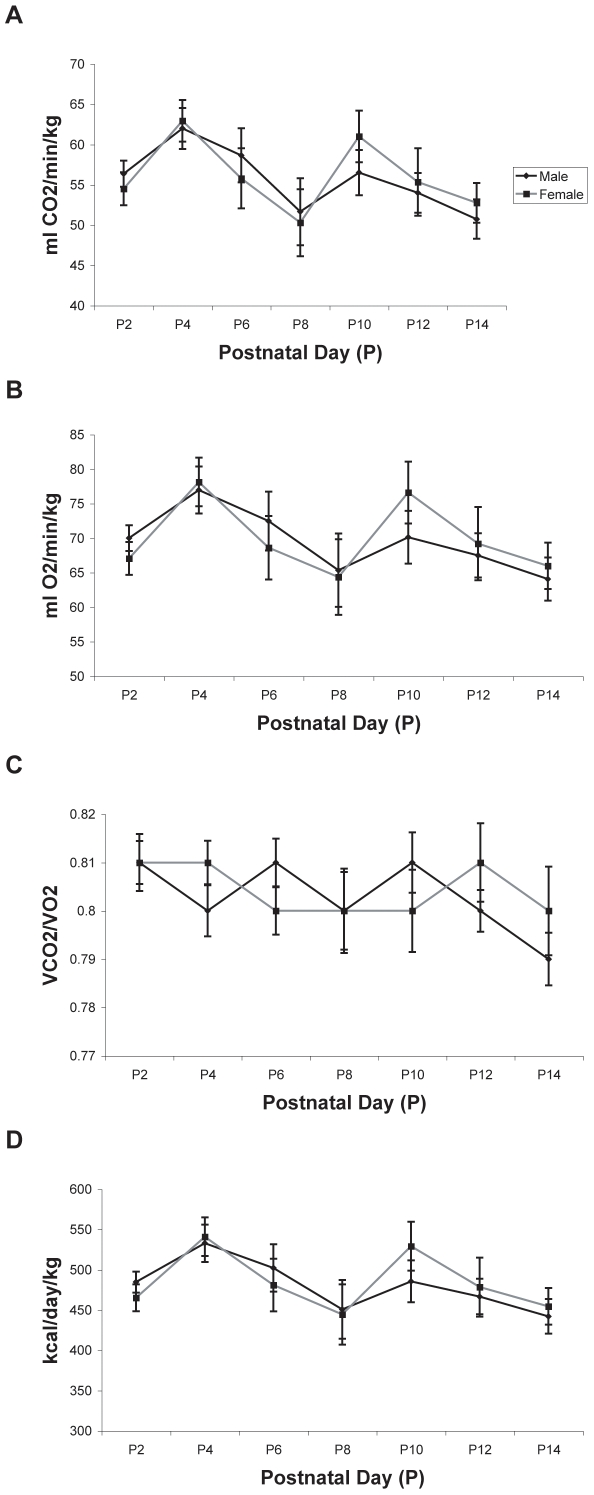
The effect of sex on metabolic parameters. Rats from small (SML), medium (MED) and large (LRG) litters were surveyed on alternate days from P2–P14 at an ambient temperature of 28°C. All data are expressed as means±SEM. No differences between groups were noted for (A) carbon dioxide production (*VCO_2_*), (B) oxygen consumption (*VO_2_*), (C) respiratory quotient (*RQ*), or (D) daily energy expenditure (*EE*).

**Figure 3:**
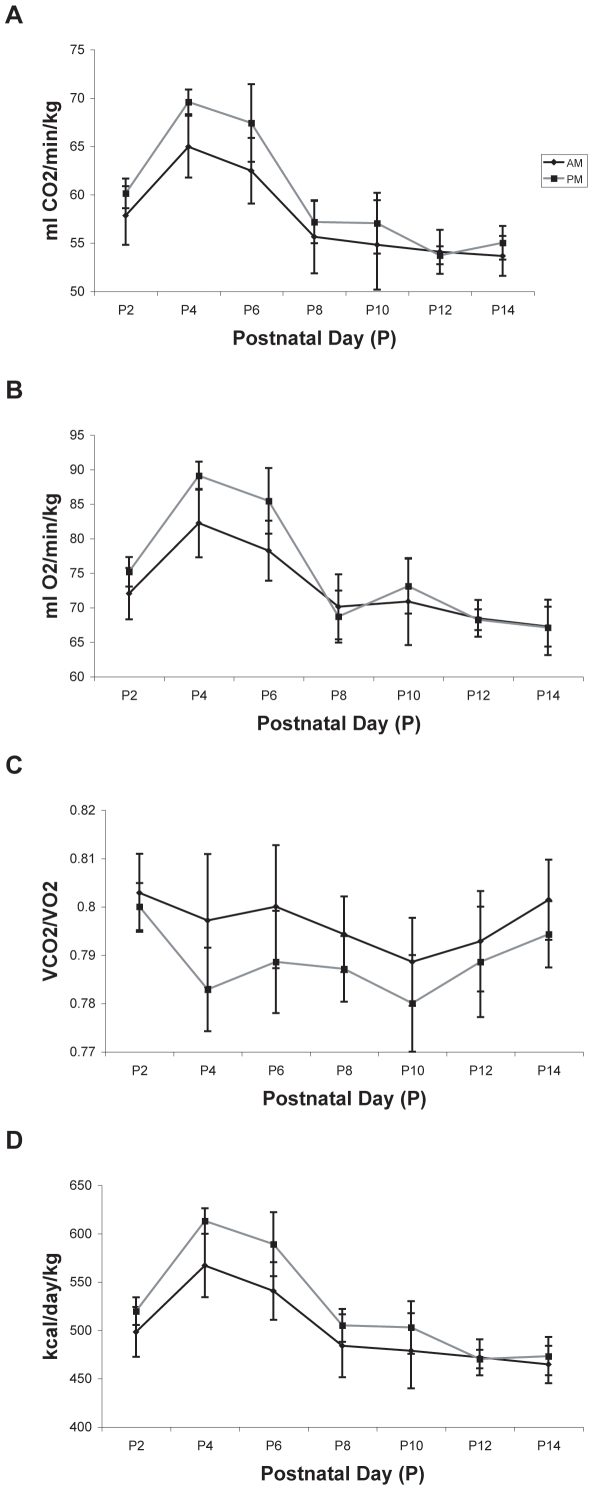
The effect of time of day (during the light phase) on metabolic parameters. Rats from small (SML), medium (MED) and large (LRG) litters were surveyed on alternate days from P2–P14 at an ambient temperature of 28°C. All data are expressed as means±SEM. No differences between groups were noted for (A) carbon dioxide production (*VCO_2_*), (B) oxygen consumption (*VO_2_*), (C) respiratory quotient (*RQ*), or (D) daily energy expenditure (*EE*).

In *Experiment 1*, we collected data from rat pups between the ages of P2 and P14 on alternate days and analyzed the impact of sex, time of day, and litter size on the expression of metabolic parameters (*VO_2_, VCO_2_, RQ, and EE*) and growth (body weight, *SR* length, and *SO* length). Our analyses using sex (Figure 2a–d) or time of day (Figure 3a–d) as between subjects factors did not reveal any significant effects on any of the metabolic variables examined, allowing us to group the animals for analysis of litter size (*Experiment 1*) and ambient temperature (*Experiment 2*) effects.

### Growth Measures

#### Body Weight

**Figure 4:**
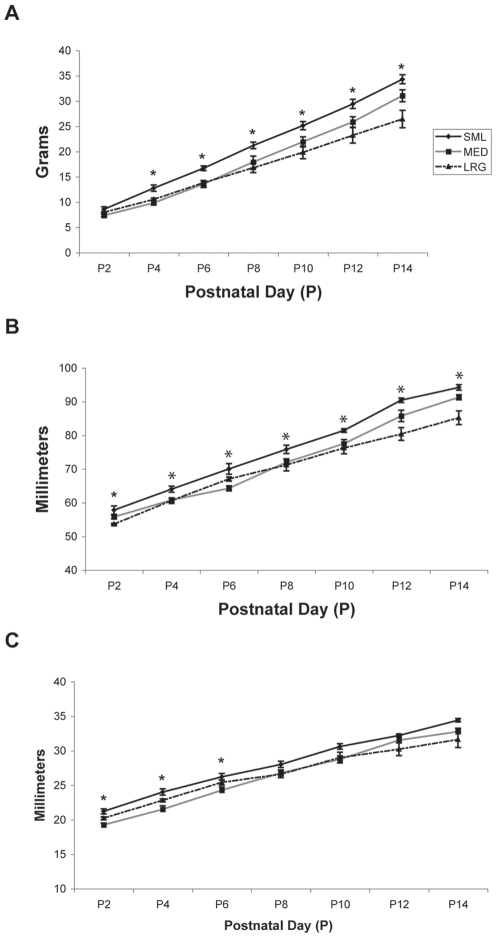
The effect of litter size on growth outcomes. Growth measures were collected for each rat subject used from small (SML), medium (MED) and large (LRG) litters at an ambient temperature of 28°C. All data are presented as means±SEM. Analysis of (A) body weight, (B) snout-to-rump (*SR*) length, and (C) snout-to-occiput (*SO*) length data revealed that rats from the SML litter showed the greatest increases in growth from P2–P14. Days in which significant differences existed between groups are noted by asterisks.

On P2, mean body weights for the *SML*, *MED*, and *LRG* groups were 8.58±0.50 g, 7.35±0.26 g, and 7.98±0.12 g, respectively (Table 1). Rats in all three litters gained weight (Figure 4a) demonstrating mean body weights of 34.3±0.88 g, 31.05±1.18 g, and 26.42±1.71 g (*SML*, *MED*, and *LRG* groups, respectively) on P14. Analysis of weight data revealed a significant litter size effect (p<0.01), significant testing day effect (p<0.01), and significant litter size×testing day interaction (p<0.01). Post hoc testing of the litter size effect revealed that the *SML* group weighed more than the *LRG* group (p<0.05). Post hoc testing of the litter size×testing day interaction revealed significant differences between P4–P14 (p values ranged from<0.01 to<0.05), with rats from the *SML* litter weighing more than rats from both *MED* and *LRG* litters on P4 and P6, and weighing more than rats from the *LRG* litter on days P8–P14 (p<0.05).

**Table 1:** Mean Growth, Metabolic, and Activity Data for Experiment 1.

Group	Category	P2	P8	P14
***Small Litter***	Weight (g)	8.58±.50	21.2±.65	34.3±.88
	SR Length (mm)	57.8±1.24	75.8±1.24	94.2±.80
	SO Length (mm)	21.2±.37	28±.45	34.4±.24
	VO2 (ml/min/kg)	77.48±4.31	66.0±4.43	69.62±2.54
	VCO2 (ml/min/kg)	61.39±3.33	50.49±3.02	54.36±2.05
	RQ	0.79±.01	0.77±.01	0.78±.00
	Energy Expenditure (kcal/day/kg)	534.59±29.57	452.21±29.68	478.89±17.56
	Activity (AU)	0.76±.24	0.43±.17	1.81±.20
***Medium Litter***	Weight (g)	7.35±.26	17.9±1.21	31.05±1.18
	SR Length (mm)	55.75±.48	72±.71	91.25±.75
	SO Length (mm)	19.25±.25	26.75±.48	32.75±.49
	VO2 (ml/min/kg)	68.40±2.32	69.14±6.92	66.48±6.9
	VCO2 (ml/min/kg)	54.18±2.04	62.28±1.49	56.37±2.41
	RQ	0.79±.01	0.81±.01	0.8±.01
	Energy Expenditure (kcal/day/kg)	471.89±16.31	535.98±15.5	477.23±31.21
	Activity (AU)	0.67±.21	0.54±.19	2.05±.49
***Large Litter***	Weight (g)	7.98±.12	16.8±.97	26.42±1.71
	SR Length (mm)	53.6±.24	71.2±1.77	85.2±2.03
	SO Length (mm)	20.2±.2	26.6±.51	31.6±1.17
	VO2 (ml/min/kg)	73.85±3.07	72.0±4.73	65.28±3.72
	VCO2 (ml/min/kg)	60.39±2.24	57.57±3.77	52.67±2.51
	RQ	0.82±.01	0.79±.00	0.81±.01
	Energy Expenditure (kcal/day/kg)	512.80±20.83	503.20±32.68	452.03±24.82
	Activity (AU)	0.69±.21	0.62±.20	2.01±.30

#### SR Length

On P2, mean body lengths for the *SML*, *MED*, and *LRG* groups were 57.8±1.24 mm, 55.75±0.48 mm, and 53.6±0.24 mm, respectively (Table 1). Rats showed an increase in *SR* length through P14 (Figure 4b, Table 1). Analysis of *SR* length data revealed a significant litter size effect (p<0.01), significant testing day effect (p<0.01), and significant litter size×testing day interaction (p<0.01). Post hoc analysis of the litter size effect showed that the *SML* group had significantly longer *SR* lengths than the *LRG* group (p<0.01). Post hoc testing of the litter size×testing day interaction revealed significant differences between P2–P14 (p values ranged from <0.01 to <0.05), with rats from the *SML* litter being longer than rats from both *MED* and *LRG* litters on P4 (p<0.05), being longer than rats from the *LRG* litter on days P2, P10, P12, and P14 (p<0.05), and being longer than rats in the *MED* litter on P6 (p<0.05).

#### SO Length

Rats showed an increase in *SO* length through P14 (Figure 4c, Table 1), reaching mean maximum lengths of 34.4±0.24 mm, 32.75±0.49 mm, and 31.6±1.17 mm (*SML*, *MED*, and *LRG* groups, respectively). Analysis of *SO* length revealed a significant litter size effect (p<0.05), significant testing day effect (p<0.01), and significant litter size×testing day interaction (p<0.01). Post hoc analysis of the litter size effect showed that the *SML* group had longer *SO* lengths than the *MED* group (p<0.062, trend) and the *LRG* group (p<0.071, trend). Post hoc testing of the litter size×testing day interaction revealed significant differences on P2–P6 (p values ranged from <0.01 to <0.05), with rats from the *SML* litter having longer *SO* lengths than rats from the *MED* litter (p<0.05).

### Metabolic Measures

Utilizing the derived inlet flow rate prediction equation and inserting the pup's weight and expected *VO_2_* from the literature, flow rates ranged from a mean of 40 ml/min for P2 rat pups to a mean of 200 ml/min for P14 rat pups (data not shown). During the acclimation period, the flow rate was finely adjusted to maintain the metabolic chamber *CO_2_* and *O_2_* concentrations within the prescribed ranges. However, the adjusted value never differed from the predicted value by more than 10 ml/min (data not shown).

### Experiment 1

#### VCO_2_

**Figure 5:**
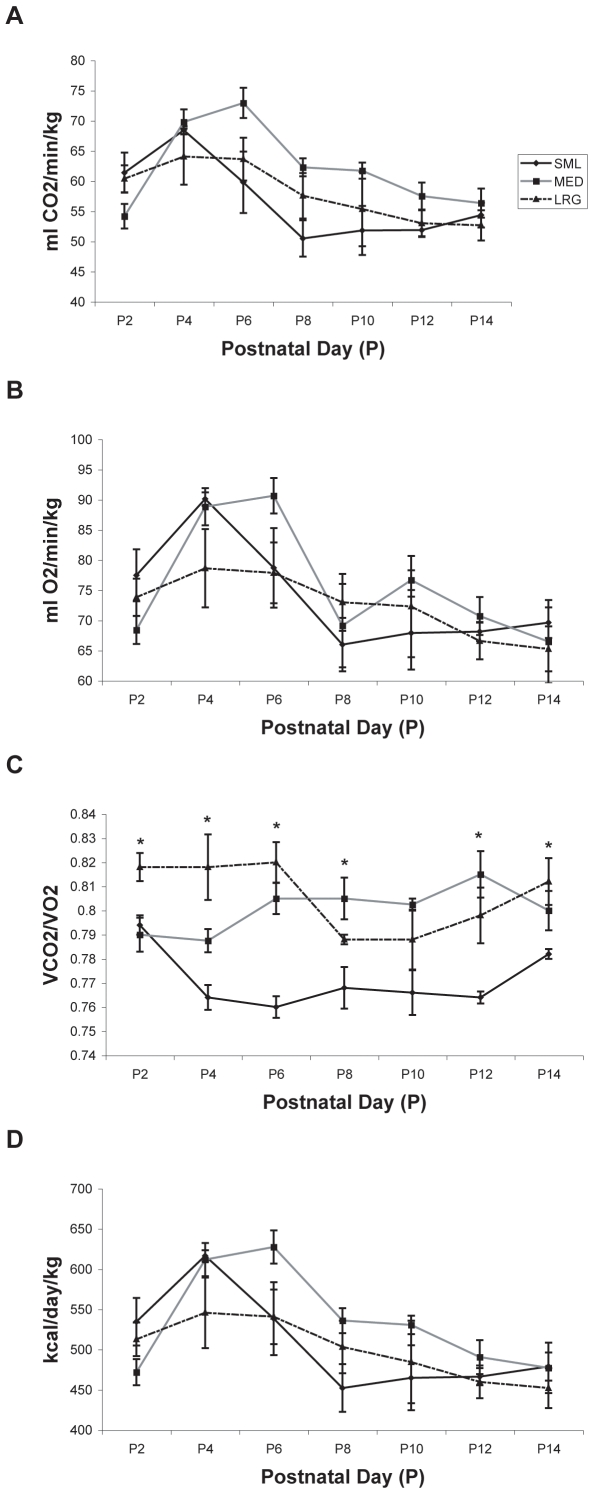
The effect of litter size on metabolic outcomes. Metabolic parameters from each rat subject in small (SML), medium (MED) and large (LRG) litters were surveyed on alternate days from P2–P14 at an ambient temperature of 28°C. All data are expressed as means±SEM. No differences between groups were noted for (A) carbon dioxide production (*VCO_2_*), (B) oxygen consumption (*VO_2_*), or (D) daily energy expenditure (*EE*). Differences between groups were observed for (C) respiratory quotient (*RQ*), with rats from the SML litter having lower *RQ's* than rats from the other litters. Days in which significant differences existed between groups are noted by asterisks.

Analysis of *CO_2_* production revealed a main effect for testing day (p<0.01), with animals showing an increase between P2–P6, and then a decline through P14 (Figure 5a). We also observed a significant testing day×litter size interaction (p<0.05). Post testing of the testing day×litter size interaction demonstrated differences on P8 (p = 0.057, trend), with rats from the *SML* litter producing less *CO_2_* when compared with the rats from the *MED* litter (p = 0.052, trend). Representative mean *VCO_2_* values on P2, P8, and P14 are shown in Table 1 for all three groups.

#### VO_2_

Analysis of *O_2_* consumption revealed a main effect for testing day only (p<0.01). During the course of metabolic data collection from P2–P14, the highest *VO_2_* values for all three litters were observed on P4 and P6, with values decreasing on P8 and remaining relatively stable through P14 in all three litters (Figure 5b, Table 1).

#### RQ

Analysis of *RQ* data demonstrated a main effect for litter size (p<0.01) and a significant testing day×litter size interaction (p<0.01, Figure 5c). Post testing of the significant litter size effect showed that the *SML* group had lower *RQ* values than the *MED* and *LRG* groups (both p<0.01). Post testing of the testing day×litter size interaction revealed differences (p<0.05) on all days except P10, with rats from the *SML* litter having lower *RQ's* than rats from both *MED* and *LRG* litters on P6 and P12 (p<0.05), rats from the *SML* litter having lower *RQ's* when compared to rats from the *LRG* litter on P4 and 14 (p<0.05), rats from the *SML* litter having lower *RQ's* than rats from the *MED* litter on P8 (p<0.05), and rats from the *SML* and *MED* litters having lower *RQ's* than rats from the *LRG* litter on P2 (p<0.05). Representative mean *RQ* values on P2, P8, and P14 are shown in Table 1 for all three groups.

#### Estimated Energy Expenditure

The Metabolism^®^ software calculated the estimated daily *EE* utilizing the *Weir equation* [12]:
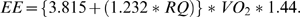



Analysis of estimated daily *EE* revealed a main effect for testing day only (p<0.01). Similar to the pattern observed for *VO_2_*, *EE* across all 3 litters slightly increased from P2 through P6, after which a decrease in *EE* values occurred at P8 and remained relatively stable through P14 (Figure 5d).

#### Activity Assessment

Representative mean gross activity scores for each group are shown in Table 1. In general, pups from all three groups did not demonstrate significant activity during the first postnatal week (spending the majority of the time sleeping in the metabolic chamber) and showed only minimal activity during the second postnatal week (a mean activity score of 2 representing movement of the forepaws only).

### Experiment 2

#### VCO_2_

**Figure 6:**
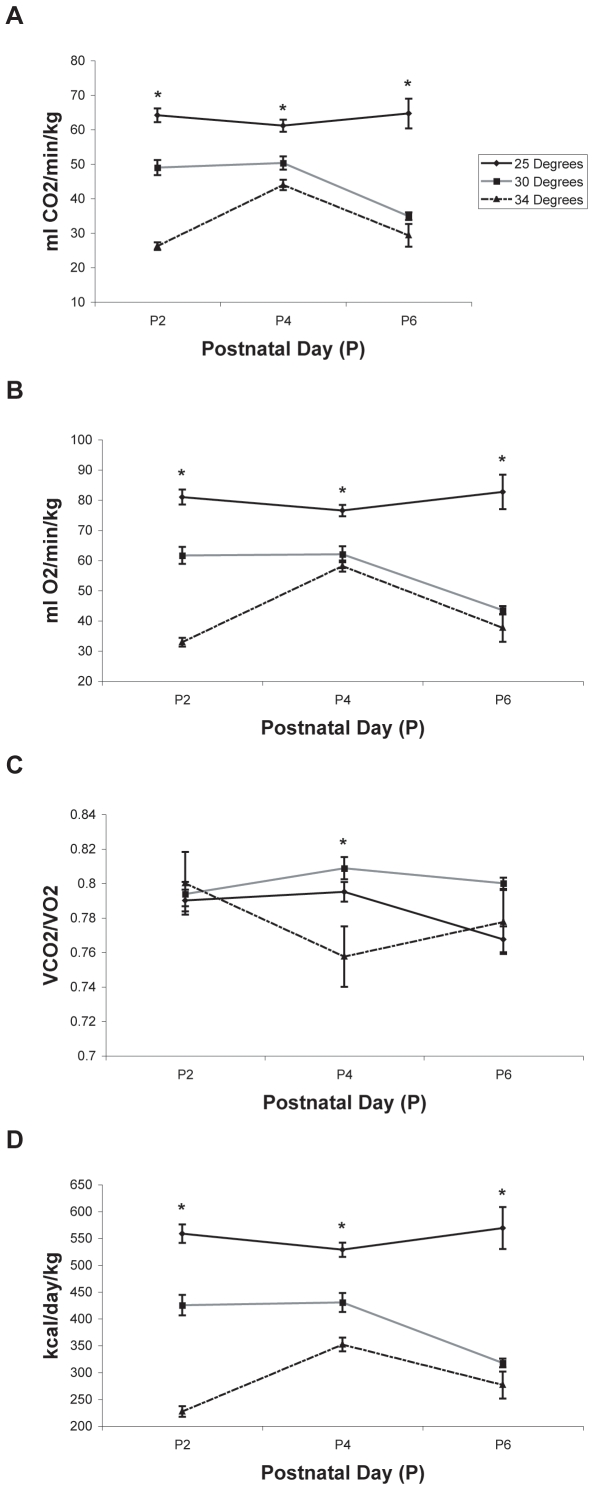
The effect of ambient temperature on metabolic outcomes. Metabolic parameters from each rat subject tested at ambient temperatures of 25°C, 30°C, and 34°C were surveyed on alternate days from P2–P6. All data are expressed as means±SEM. Differences between groups were observed for (A) carbon dioxide production (*VCO_2_*), (B) oxygen consumption (*VO_2_*), (C) respiratory quotient (*RQ*), and (D) daily energy expenditure (*EE*). Rats tested at 25°C demonstrated the highest *VCO_2_*, *VO_2_*, and *EE* values, while rats tested at 34°C demonstrated the lowest values in the same parameters. Days in which significant differences existed between groups are noted by asterisks.

Mean *VCO_2_* values ranged from 26.17±1.12 ml/min/kg on P2 in the *34°C* group to 64.63±4.31 on P6 in the *25°C* group (Figure 6a). Analysis of *CO_2_* production revealed a main effect for ambient temperature (p<0.01), a main effect for testing day (p<0.05), with animals from the *30°C* and *34°C* groups showing a decrease in *CO_2_* production on P6 (Figure 6a), and a significant testing day×ambient temperature interaction (p<0.01). Post testing of the significant ambient temperature effect revealed significant differences between all groups (p<0.01), with *VCO_2_* values ordered as follows: *25°C* group>*30°C* group>*34°C* group. Post testing of the testing day×ambient temperature interaction revealed significant differences in *CO_2_* production between all three groups from P2–P4 (p<0.01).

#### VO_2_

Mean *VO_2_* values ranged from 32.84±1.46 ml/min/kg on P2 in the *34°C* group to 82.65±5.71 ml/min/kg on P6 in the *25°C* group (Figure 6b). Analysis of *O_2_* consumption revealed a main effect for ambient temperature (p<0.01), a main effect for testing day (p<0.05), with animals from the *30°C* and *34°C* groups showing a decrease in *O_2_* consumption on P6, and a significant testing day×ambient temperature interaction (p<0.05). Post testing of the ambient temperature effect revealed significant differences between all groups (p<0.01) with the *25°C* consuming the most *O_2_*, followed by the *30°C* group, and the *34°C* group consuming the least amount of *O_2_*. Post testing of the testing day×ambient temperature interaction revealed significant differences in *O_2_* consumption between all three groups from P2–P4 (p<0.01).

#### RQ

The lowest (.76±.02) and highest (.81±.01) mean *RQ* values were exhibited on P4 by the *34°C* group and the *30°C* group, respectively (Figure 6c). Analysis of *RQ* data demonstrated a main effect for ambient temperature (p<0.01), a main effect for testing day (p<0.05), with the *RQ* decreasing in the *25°C* and *34°C* groups from P2–P6, and a significant testing day×ambient temperature interaction (p<0.01). Post testing of the significant ambient temperature effect revealed that rats in the *30°C* group exhibited the highest RQ values when compared to rats in the *25°C* and *34°C* groups (p<0.01 respectively). Post testing of the testing day×ambient temperature interaction revealed significant differences between groups on P4, with rats in the *25°C* and *34°C* groups demonstrating a lower *RQ* than the rats in the *30°C* group (p<0.05).

#### Estimated Energy Expenditure

The lowest mean energy expenditure (226.87±9.91 kcal/day/kg) was noted in the *34°C* group on P2 and the highest mean energy expenditure (568.78±38.96 kcal/day/kg) was observed in the *25°C* group on P6 (Figure 6d). Analysis of estimated daily *EE* revealed a main effect for ambient temperature (p<0.01), a main effect for testing day (p<0.05), with animals from the *30°C* and *34°C* groups showing a decrease in *EE* on P6, and a significant testing day×ambient temperature interaction (p<0.01). Post testing of the significant ambient temperature effect revealed differences between all groups (p<0.01) with rats in the *25°C* group expending the most energy, followed by rats in the *30°C* group, and rats in the *34°C* group expending the least energy. Post testing of the testing day×ambient temperature interaction revealed significant differences in *EE* between all three groups from P2–P4 (p<0.01).

## Discussion

Our interdisciplinary team developed a novel indirect calorimetry system to study the metabolic parameters of neonatal rodent pups. To validate the system, we conducted two experiments to evaluate its ability to detect differences in metabolic parameters from known or expected sample outcomes. The results of the present study demonstrated that the prototype Panlab Oxylet system was capable of collecting and analyzing respiratory gases to determine metabolic parameters in individual neonatal rat pups under varied natural and environmental conditions. Consistent with previous studies, we demonstrated that rats from small litters have increased weight gain during the preweaning period [6]. We extended these findings by demonstrating that rats from small litters also show the greatest body and head length changes over the first two weeks of development. Our lab is interested in different variables that impact early growth and here tested a new system that will help elucidate the impact of metabolic parameters on neonatal growth. We showed that litter size and ambient temperature influence metabolic parameters in neonatal rats and that the Oxylet system was capable of detecting these differences.

In *Experiment 1*, the *RQ* value for the *SML* group was close to 0.80 on P2 and then remained around 0.76 from P4–P12, increasing slightly to 0.78 on P14, in contrast to the higher *RQ* values exhibited by the *MED* and *LRG* groups during the same timeline. Fiorotto et al [6] demonstrated that litter size significantly affects the dam's milk fat and protein content, which suggests that litter size would influence a neonatal rat's *RQ*. A litter size of 4 pups is associated with an increased fat concentration in breast milk from P6–P10 and P10–P14 compared to breast milk from dams with litters of 10 or 16. The small litter size is also associated with a significantly lower breast milk protein content from P4–P6, P6–P10, and P10–P14 compared to breast milk from dams with larger litters. Our finding of a reduced *RQ* value in the *SML* group is consistent with this and demonstrates that the Oxylet system is capable of detecting this expected result. From P6–P14, the *RQ* values for the *MED* and *LRG* groups clustered around 0.79–0.80 and were in agreement with that reported by previous investigators for similar litter sizes [8], [13], further supporting the system's sensitivity.

Early studies documented metabolic parameters in rat pups utilizing technology that included *CO_2_* traps and infrared *CO_2_* analyzers in conjunction with polarographic *O_2_* analyzers [4], [8]. Our study is the first to demonstrate indirect calorimetry technology the combines thermal mass flow sensors, laser diode absorption *O_2_* analyzers, and infrared spectroscopy *CO_2_* sensors to assess metabolic outcomes in rat pups as young as 2 days. Several authors have reported *VO_2_* values ranging from 30–57 ml/min/kg at an ambient temperature of 30°C in rat pups 5–6 days old [4], [7]. We found a mean *VO_2_* of ∼43 ml/min/kg in P6 rats at the same ambient temperature that are in agreement with these previously reported values, further validating the Oxylet system. Our finding of a mean *VO_2_* of ∼37 ml/min/kg in P6 rats at an ambient temperature of 34°C is in close agreement with Planche et al's [8] reported *VO_2_* of 39 ml/min/kg in 7-day-old rat pups at an ambient temperature of 33°C.

Together, these data demonstrate that utilizing the derived *F_e_* equation reliably and consistently satisfied the experimental constraints of: 1) maintaining the difference between ambient and chamber [*O_2_*] and [*CO_2_*] within a range of 0.5–1.0%, 2) maintaining the chamber [*CO_2_*] below 1.0% to avoid inducing hyperventilation in response to hypercapnea, and 3) slowing down the sampling process to allow equilibration of respiratory gases within the chamber so that small changes created by neonatal rat pups as young as 1–2 days old could be measured. By employing the derived *F_e_* equation, it was possible to estimate inlet flow rates that yielded reliable results on the first attempt and minimized trial and error approaches that would have prolonged the pup's separation from the mother and added stress as a confounding variable. This prediction equation can thus be incorporated into the existing Panlab Metabolism^®^ software in the future, improving its ease of use.

This technology shows great promise for use in rodent models of human disease states, where phenotyping neonatal rodent pups is critical for studying the developmental sequelae of experimental manipulations or therapeutic interventions. Furthermore, since mice attain comparable weight and metabolic activity to 2-day-old rat pups on P7, the system shows great potential in the ability to measure metabolic variables in transgenic and knockout models during the second week of postnatal life. Further studies are warranted to assess its plausible use in mice younger than P7.

The results of this study demonstrated that 1) the Panlab Oxylet system in it's modified form could reliably assess early postnatal metabolism in rats as young as 2 days of age, 2) early postnatal resting metabolism is influenced by litter size and ambient temperature, but not by sex or time of day (during the light phase), and 3) the modified system should be suitable for studying the metabolism of early postnatal mice during the second postnatal week of life. This system will prove valuable for enhancing our understanding of early postnatal metabolism in rodent models of human disease states.
